# Characterization of magnesium requirement of human 5'-tyrosyl DNA phosphodiesterase mediated reaction

**DOI:** 10.1186/1756-0500-5-134

**Published:** 2012-03-09

**Authors:** Sanjay Adhikari, Soumendra K Karmahapatra, Tejaswita M Karve, Sanjona Bandyopadhyay, Jordan Woodrick, Praveen V Manthena, Eric Glasgow, Stephen Byers, Tapas Saha, Aykut Uren

**Affiliations:** 1 Department of Oncology, Lombardi Comprehensive Cancer Center, Georgetown University Medical Center, Washington, DC 20057, USA; 2 Department of Biochemistry and Molecular and Cellular Biology, Lombardi Comprehensive Cancer Center, Georgetown University Medical Center, Washington, DC 20057, USA

## Abstract

**Background:**

Topo-poisons can produce an enzyme-DNA complex linked by a 3'- or 5'-phosphotyrosyl covalent bond. 3'-phosphotyrosyl bonds can be repaired by tyrosyl DNA phosphodiesterase-1 (TDP1), an enzyme known for years, but a complementary human enzyme 5'-tyrosyl DNA phosphodiesterase (hTDP2) that cleaves 5'-phosphotyrosyl bonds has been reported only recently. Although hTDP2 possesses both 3'- and 5'- tyrosyl DNA phosphodiesterase activity, the role of Mg^2+ ^in its activity was not studied in sufficient details.

**Results:**

In this study we showed that purified hTDP2 does not exhibit any 5'-phosphotyrosyl phosphodiesterase activity in the absence of Mg^2+^/Mn^2+^, and that neither Zn^2+ ^or nor Ca^2+ ^can activate hTDP2. Mg^2+ ^also controls 3'-phosphotyrosyl activity of TDP2. In MCF-7 cell extracts and de-yolked zebrafish embryo extracts, Mg^2+ ^controlled 5'-phosphotyrosyl activity. This study also showed that there is an optimal Mg^2+ ^concentration above which it is inhibitory for hTDP2 activity.

**Conclusion:**

These results altogether reveal the optimal Mg^2+ ^requirement in hTDP2 mediated reaction.

## Introduction

The topoisomerase II (TopII) family is an important class of topoisomerases whose activity consists of reaction cycles of DNA binding, DNA cleavage, DNA strand passage, and religation of the cleaved DNA. DNA cleavage involves formation of a reversible intermediate consisting of an active site tyrosine residue that forms a phosphotyrosyl linkage with DNA. One major difference between topoisomerase I (TopI) and TopII is that TopII produces 5' DNA-protein crosslinks simultaneously in both strands whereas Top1 produces 3' DNA-protein crosslinks in one strand [1-6]. Drugs targeting these enzymes act by preventing the religation of DNA and produces protein-DNA covalent complexes along with single- and double strand breaks [7,8]. However, the repair of TopII-DNA complexes is poorly understood. Recently, a human 5'-tyrosine phosphodiesterase (hTDP2) has been identified for the excision of TopII-DNA adducts [9,10]. Previously, TDP2 was known as TTRAP (TRAF and TNF receptor-associated protein), a protein of unknown function and a putative member of the Mg^2+^/Mn^2+^-dependent phosphodiesterase superfamily, with the DNA repair protein apurinic/apyrimidinic (AP) endonuclease-1 (APE-1, also known as APEX1) being its closest relative. hTDP2 possesses both 3' phosphotyrosyl and 5' phosphotyrosyl activity. Knockdown/knockout of TDP2 in A549 and DT40 cells increased sensitivity to the TopII targeting agent etoposide but not to the TopI targeting agent camptothecin (CPT). The 5'-tyrosyl DNA phosphodiesterase activity of hTDP2 can enable the repair of TopII-induced double strand breaks (DSBs) without the need for nuclease activity, because it creates a "clean" DSB with 5'-phosphate termini and a 3'-hydroxyl group. These "clean" DSBs are religatable by DNA ligase, providing an opportunity for error free repair [9,10]. hTDP2 may thus provide an "error-free" mechanism for direct end-joining of TopII-induced DSBs. This is different from currently established mechanisms for DSB repair, which involve structure-specific nucleases [11].

In the present study, we demonstrate that absolutely no product was formed in the hTDP2-mediated reaction in the absence of Mg^2+^, even with a higher concentration of hTDP2 but there is an optimal Mg^2+ ^concentration above which it is inhibitory for hTDP2 activity. Like many other Mg^2+^-dependent enzymes, hTDP2 showed similar activation by Mn^2+^.

## Results

### Purification of hTDP2

**Figure 1:**
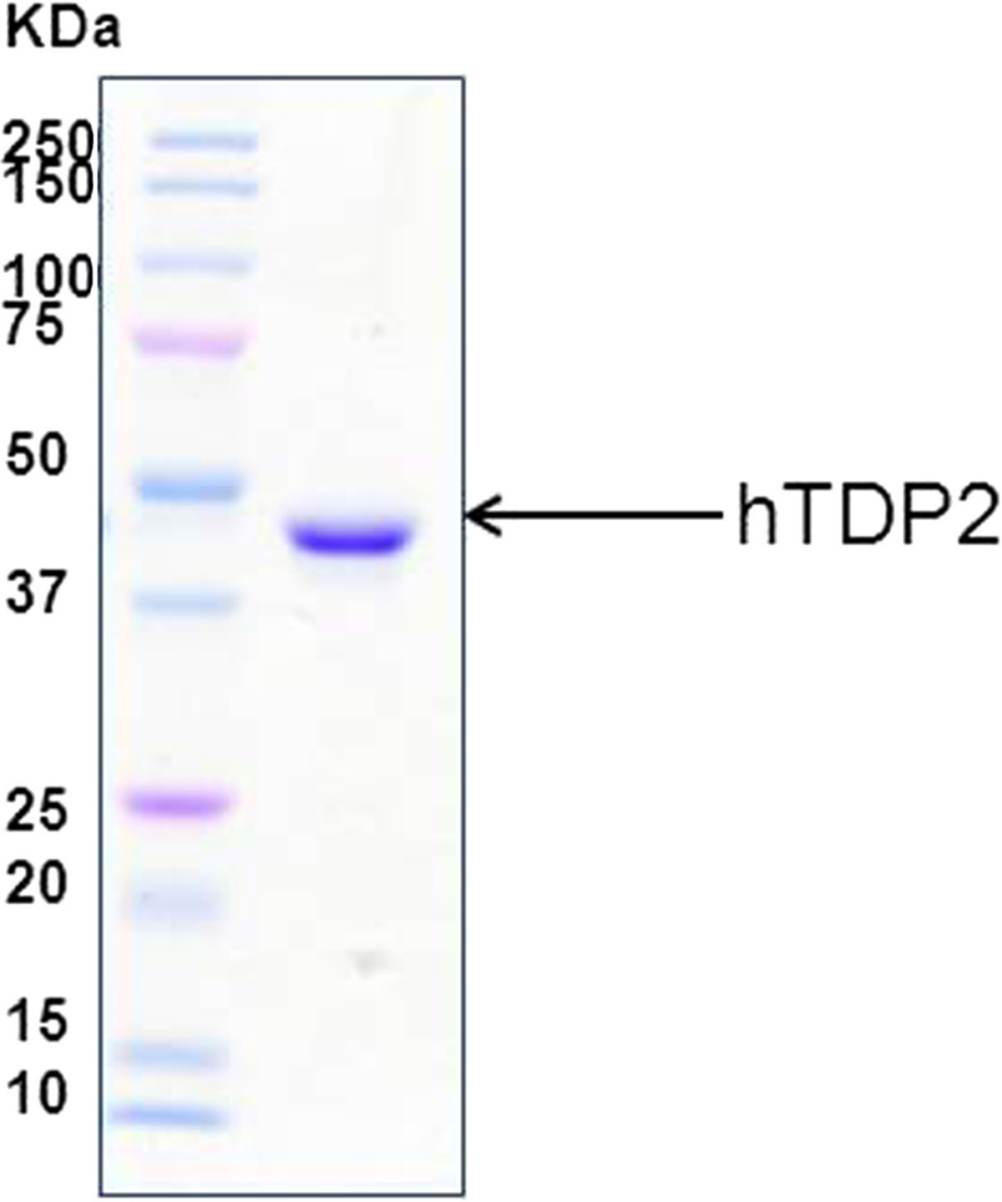
**Purification of hTDP2**. SDS-PAGE of purified hTDP2 proteins after coomassie staining. The details of the purification are described in "Materials and Methods".

The hTDP2 was purified using the methods described in "Materials and Methods." The best fraction of hTDP2 protein was used, which is more than 95% pure electrophoretically (Figure 1). We obtained 7.5 mg total protein from 2 liter *E.coli*. culture with a concentration of 27 μM.

### Effect of Mg^2+ ^and other divalent ions on hTDP2 activity

**Figure 2:**
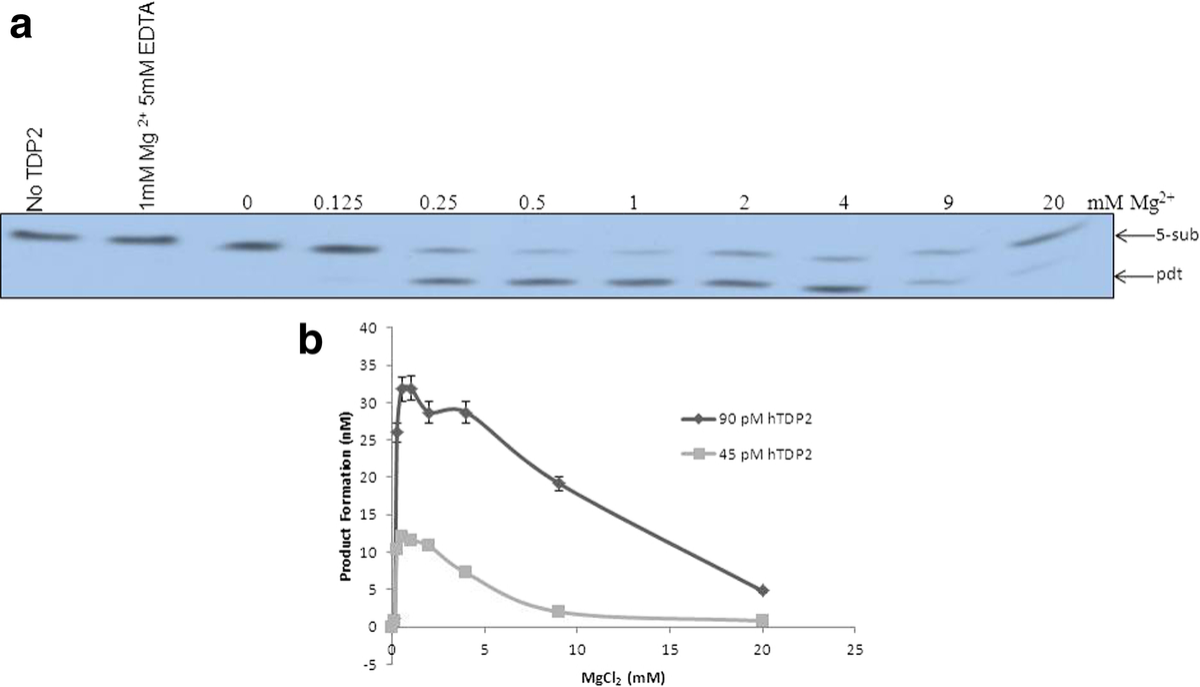
**Modulation of product formation by Mg^2+ ^in hTDP2-mediated 5'-phosphotyrosyl bond cleavage reaction**. **(A) **hTDP2 (90 pM) was reacted with a 5-sub (40 nM) at 37°C for 7 min. The details of the reaction conditions are described in "Materials and Methods." **(B) **Data obtained in Panel A and a separate set of experiments using 45 pM TDP2 under similar reaction conditions as in Panel A were plotted. "Pdt" represents respective formed product in this and other figures in the paper.

**Figure 3:**
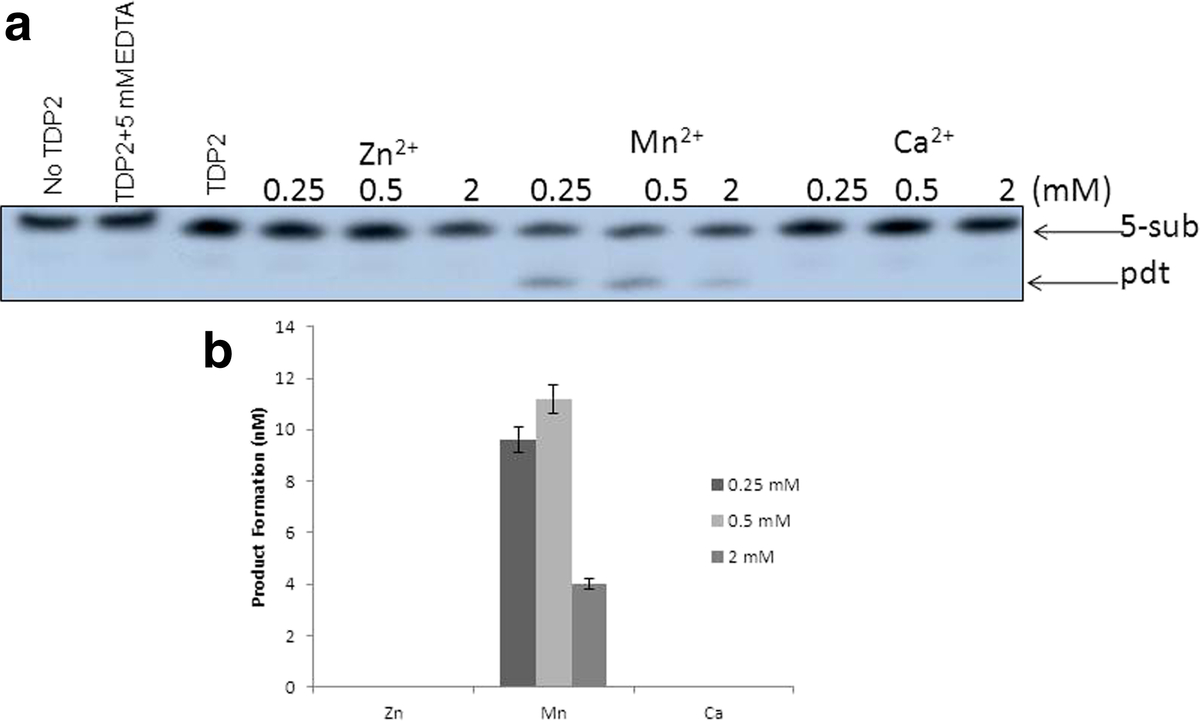
**Effect of different divalent metals on hTDP2-mediated 5'-phosphotyrosyl bond cleavage reaction**. **(A) **TDP2 (90 pM) was reacted with 5-sub (40 nM) under conditions similar to those described in Figure **2 **with the exception of the addition of different divalent metal ions. **(B) **Data obtained in Panel A was plotted. Data represent mean values with standard error derived from three independent experiments.

**Figure 4:**
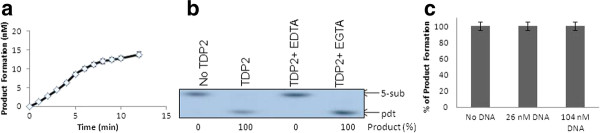
**Characterization the effect of Mg^2+ ^in hTDP2-mediated 5'-phosphotyrosyl bond cleavage reaction**. (**A**) Time kinetics experiment was performed using 45 pM TDP2 and 20 nM of the 5-sub substrate for 0-12 mins incubating at 37°C. **(B) **hTDP2 (9 nM) was reacted with 5-sub (50 nM) at 37°C for 7 min in the absence/presence of EDTA and EGTA. **(C) **hTDP2 (90 pM) was reacted with a 5-sub substrate (40 nM) at 37°C for 7 min in the presence of different concentrations of DNA. Data represent mean values with standard error derived from three independent experiments.

**Figure 5:**
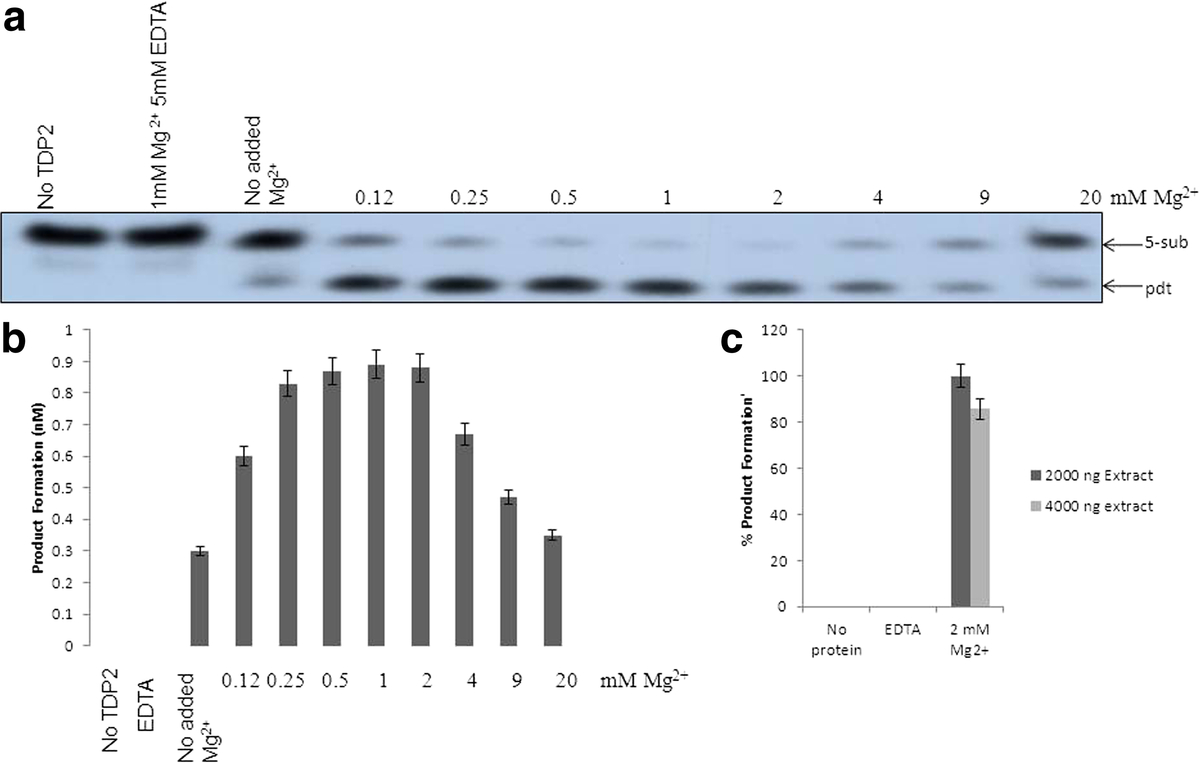
**Modulation of 5'-phosphotyrosyl bond cleavage by Mg^2+ ^in MCF-7 whole cell extract and zebrafish embryo extract**. 1 μg of MCF7 whole cell extract was reacted with 5-sub (1 nM) for 10 minutes under conditions described in "Material & Methods." **(A) **Data obtained in Panel A was plotted **(B) **2 μg and 4 μg of zebrafish embryo extract reacted with 5-sub (1 nM) **(C) **Data represent mean values with standard error derived from three independent experiments.

**Figure 6:**
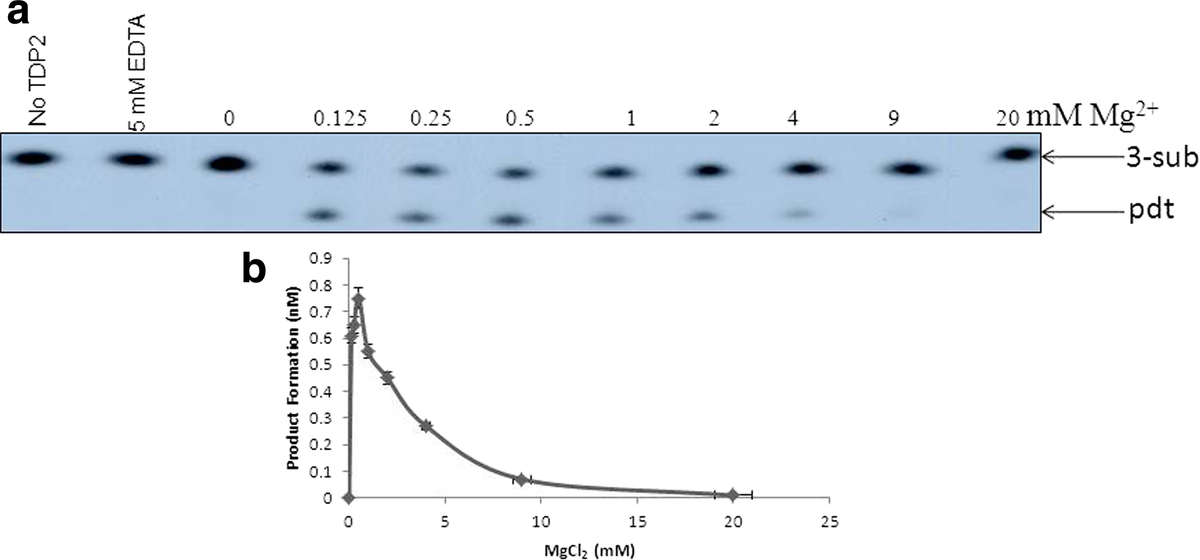
**Modulation of product formation by Mg^2+ ^in hTDP2-mediated 3'-phosphotyrosyl bond cleavage reaction**. **(A) **TDP2 (180 nM) was reacted with 3-sub (1 nM) for 20 minutes under conditions similar to those shown in Figure **1**. **(B) **Data obtained in Panel A was plotted. Data represent mean values with standard error derived from three independent experiments.

The activity of purified TDP2 was measured in the presence of various (0-20 mM) MgCl2 concentrations using 5-sub (details in the "Methods" section) as the substrate (Figure 2A, B), and it was observed that Mg^2+ ^is absolutely required for product formation (0.5-1 mM is the optima). Next, we tested the effect of other divalent metals on hTDP2 mediated enzymatic reaction. Like many other Mg^2+ ^-dependent enzymes hTDP2 showed activation in presence of Mn^2+ ^but with less efficiency than Mg^2+^, whereas in the presence of Zn^2+^or Ca^2+ ^there is no hTDP2 mediated activity (Figure 3A, B). So it can be concluded that Mg^2+^/Mn^2+^- mediated activity is specific. For further investigation, we performed a detailed study with Mg^2+^, as it is more potent to activate hTDP2 (Figures 2 and 3). Time kinetics experiments with 1 mM Mg^2+ ^showed that hTDP2 has extremely fast kinetics (Figure 4A) and that the product formation is linear for the range of 0-7 minutes [R2 < 0.99]. In the presence of excess EDTA, no product formation was observed (Figure 4B). EGTA cannot prevent the product formation by Mg^2+ ^(Figure 4B). EGTA, a specific chelator for Ca^2+^, has a significantly higher affinity for Ca^2+ ^than for Mg^2+^, and thus the results are in accordance with expectations. Based on the findings that EDTA, not EGTA, can reverse the product formation, it appears that the Mg^2+^-mediated product formation of TDP2 is reversible and specific. Mg^2+ ^has been shown to modulate the double-helix structure of DNA [12]. Mg^2+ ^may be modifying the DNA structure by direct binding and thus modulating the binding (and activity) of hTDP2 to the DNA substrate. We tested this possibility by adding an increasing amount of double stranded undamaged oligonucleotide without tyrosyl. Results showed that unlike EDTA, DNA could not modulate hTDP2 activity from Mg^2+^-mediated product formation (Figure 4C). If Mg^2+ ^binds to DNA and thus modulates the activity by changing DNA structure, then excess amount of additional double stranded DNA should compete with the substrate DNA for Mg^2+ ^binding. This would prevent Mg^2+ ^from binding to the substrate and modulate product formation, but this is not the case. Thus our results imply DNA binding of Mg^2+ ^is not responsible for its role in product formation in the TDP2 reaction. Then we checked the effect of Mg^2+ ^and EDTA on 5'- tyrosyl DNA phosphodiesterase activity in MCF-7 whole cell extract and found absolutely no product formation in the presence of excess EDTA; although without additional Mg^2+ ^(0 mM Mg^2+ ^and 0 mM EDTA), cell extracts showed some product formation, indicating the presence of residual metal ions in the extract (Figure 5A, B). To evaluate whether or not this property is species dependent, we used de-yolked zebrafish embryo extract to determine the role of Mg^2+ ^in zebrafish 5'- tyrosyl DNA phosphodiesterase activity and found no product formation in the absence of Mg^2+ ^(Figure 5C). We consider that it is highly possible that TDP2 is responsible for the activity shown by the cell extract and the embryo extract, but it is possible that other unknown proteins of similar type may also be present. Since hTDP2 has 3'- tyrosyl DNA phosphodiesterase activity, we also tested the Mg^2+^-dependency of hTDP2's excision activity for 3'- tyrosyl DNA substrates, and a similar pattern was observed (Figure 6A, B).

## Discussion

DNA topoisomerases carry out their reactions by generating transient covalent phosphotyrosine intermediates with DNA. However, a variety of agents, including some anti-cancer and anti-bacterial agents, are able to trap topoisomerases while they are covalently bound to DNA [1,2]. Trapping of topoisomerases leads to DNA damage that includes strand breaks and protein covalently bound to DNA. Strand breaks can be repaired by different repair pathways, such as homologous recombination and non-homologous end-joining. The removal of protein covalently bound to DNA is an interesting and unusual challenge for cells.

Topoisomerase-mediated DNA damage can be repaired by two major classes of enzymatic activities: nucleases and some specific phosphodiesterases. The first identified phosphodiesterase enzyme that could carry out this reaction was tyrosyl DNA phosphodiesterase 1 (TDP1). Mammalian TDP1 is specific for 3'- phosphotyrosyl linked peptide and lacks the ability to process 5' phosphotyrosyl- linked peptides [13,14]. It was anticipated that other mammalian enzymes might process 5' phosphotyrosyl "adducts". Recently Ledesma and colleagues have found a protein, TTRAP, which has this property and named this enzyme TDP2 [9]. Along with its probable neuroprotective role and role in carcinogenesis, hTDP2 may be a very important target for chemotherapy as it repairs the toxic adducts produced by clinical chemotherapeutic agents like etoposide [10]. Before being identified, hTDP2 was a putative member of the Mg^2+^/Mn^2+^-dependent phosphodiesterase superfamily, with the DNA repair protein APE-1 being its closest relative. Magnesium is an absolute requirement for endonucleolytic activity of APE1 [15-19].

Mg^2+ ^affects repair enzymes in many different ways. Depending on the condition, it can be inhibitory to an enzyme like N-methylpurine DNA glycosylase (MPG) [20]. Mg^2+ ^has also been shown to stimulate the turnover of thymine DNA glycosylase (TDG) [21]. For base discrimination, human endonuclease-III (hNTH1) also depends strongly on Mg^2+ ^[22]. However, most importantly Mg^2+ ^acts as a cofactor for many enzymes involved in oxidative phosphorylation, nucleic acid and protein synthesis, and mitotic activity of normal cells. Addionally, TopII also requires Mg^2+ ^for its activity [23].

In this study we have shown that hTDP2 has robust 5' phosphotyrosyl bond cleaving activity and that Mg^2+ ^is essential for it. We show that hTDP2 has optimum activity in a broad concentration range of Mg^2+ ^(0.25-2 mM), but at very high concentrations (10-20 mM) the activity is inhibited (Figure 2). So, experiments of hTDP2 activity containing 10-20 mM Mg^2+ ^underestimate its enzyme efficiency [9,10]. Mg^2+^-mediated inhibition can be due to potential non-specific binding of excess Mg^2+ ^to hTDP2. It is also possible that hTDP2, like APE1, has two possible binding sites for Mg^2+ ^[17,19]. One binding site may have more affinity towards Mg^2+ ^and may be important for activity. Another binding site with weak Mg^2+ ^affinity is important for inhibition of product formation. In that case, Mg^2+ ^binding can regulate hTDP2 activity. Notably, intranuclear Mg^2+ ^concentration is highly variable, and depending on conditions, may vary up to 75 mM. It is reported that some tumor cells contain higher Mg^2+ ^amount in the nucleus compared to normal cells [24,25].

Alternatively, one can predict that along with the protein, Mg^2+ ^can bind to the DNA and change the DNA conformation to the active (product forming) conformation. To rule out this possibility, we executed the experiment with additional DNA of the same size and sequence without the phosphotyrosyl residue and found that it did not have any effect on the reaction (Figure 4C). Moreover, one can predict that, other than Mg^2+^, the anion (Cl^-^) may have an effect on hTDP2's activity. We used 50 mM KCl concentration in all our reactions for this study. Thus, further addition of 1 mM MgCl2 should increase negligible Cl^- ^compared with the existing Cl^- ^ions. More importantly if the anion (Cl^-^) is crucial for hTDP2 reaction, one could expect a similar effect since all cationic metals used in this study have the same anionic counterpart (Figure 3). However, our results demonstrate that this not the case. On the other hand, like many other Mg^2+^-dependent enzymes, hTDP2 is active in the presence of Mn^2+^, whereas in the presence of Zn^2+^or Ca^2+ ^there is no hTDP2-mediated activity, showing that this Mg^2+^/Mn^2+^-mediated activity is specific. Also, there is absolutely no product formation in the presence of EDTA, even under very high substrate and protein concentrations (Figure 4B). The fact that absolutely no product is formed strongly indicates that the product formation is absolutely dependent on Mg^2+^. This effect of Mg^2+ ^is similar in zebrafish embryo extract and in MCF7 cell extract, indicating that it is true across species.

## Conclusion

We have demonstrated here that Mg^2+ ^is essential for the catalytic activity of hTDP2, but there is an optimal Mg^2+ ^concentration above which it is inhibitory for hTDP2 activity. Thus, our results elucidate the optimal concentration Mg^2+ ^requirement for hTDP2 activity.

## Methods

### Cloning and purification of recombinant human TDP2 (hTDP2)

An expression construct encoding hTDP2 was prepared by ligating a PCR product containing the hTDP2 coding sequence at *Nde*I and *BamH*I sites of the pET15b vector. PCR was carried out using a cDNA construct of TDP2 bought from Open Biosystems (Huntsville, AL) as the template and primers (5'-CATATGGAGTTGGGAGTTGC-3' and: 5'-GGATCCTTACAATATTATATCTAAGTTGCACAGAAG-3'.) The primers allowed the introduction of *Nde*I and *BamH*I sites at 5' and 3' ends, respectively.

The PCR products were then subcloned in TA cloning vector, digested with *Nde*I and *BamH*I, and subcloned into expression vector pET15b at *Nde*I/*BamH*I sites, allowing us to express hTDP2 protein. The identity of the construct was confirmed by DNA sequencing. hTDP2 was overexpressed in *E.coli *BL21(DE3) cells and purified to near electrophoretical homogeneity as follows. *E. coli *BL21(DE3) carrying the construct with hTDP2 was grown in magnificent broth (MacConnell Research, CA) at 37°C until the absorbance at 600 nm reached 1. The culture was cooled to 25°C and, after the addition of IPTG to 1 mM, was grown at 25°C for 16 h prior to chilling to 0°C. All subsequent procedures were carried out at 0°C. After the bacteria were harvested by centrifugation, they were resuspended in Buffer A (40 mM Tris-HCl pH 7.5, 10% glycerol, 300 mM NaCl, 0.05% Tween -20, 1 mM DTT, 30 mM Imidazole.) The cells were then lysed as described previously [26]. The lysate was clarified by centrifugation at 27000 rpm (rotar: Beckman Coulter, 50.2 Ti) for 30 min and the supernatant was applied to a 1 ml Ni-NTA column, which was pre-equilibrated with Buffer A. The column was washed with 10-column volumes of Buffer A and eluted with a gradient of 0-100% of Buffer B (Buffer A plus 500 mM Imidazole) in Buffer A. The best fractions containing hTDP2, tested by SDS-PAGE, were pooled and dialyzed against storage Buffer C (25 mM Tris-Cl, pH 7.5, 100 mM NaCl, 1 mM DTT, 10% glycerol). Protein concentration was measured by UV absorbance at 280 nm using an extinction coefficient of 43,150 (M^-1 ^cm^-1^). After confirming its identity by mass spectrometry, the protein was stored at -80°C in aliquots for future use.

## Oligonucleotide substrates preparation

3' -phosphotyrosyl and 5'-phosphotyrosyl- containing oligonucleotide (3-sub and 5-sub) was made as described previously [27].

### 5'- and 3-' tyrosyl DNA phosphodiesterase activity assay of hTDP2

The purified hTDP2 proteins were individually incubated with labeled double stranded 5'-phosphotyrosyl oligonucleotide substrate (5-sub) in the presence of different divalent metal ions/EDTA/EGTA for 7 min at 37°C in an assay buffer (50 mM Tris-Cl, pH 7.5, 1 mM DTT, 50 mM KCl and 100 μg/ml BSA) in a total volume of 20 μl. For time kinetics experiments, 45 pM of hTDP2 was incubated with 20 nM of substrate in the presence of the assay buffer indicated previously with addition of 1 mM MgCl2. The reaction was stopped by inactivating the enzyme at 80°C for 5 min.

For the 3'- activity assay, 1 nM labeled double stranded 3'-phosphotyrosyl oligonucleotide substrate (3-sub) was incubated with hTDP2 protein (180 nM) in the presence of different concentrations of MgCl2/EDTA for 20 min at 370 C in an assay buffer (50 mM Tris-Cl, pH 7.5, 1 mM DTT, 50 mM KCl and 100 μg/ml BSA) in a total volume of 20 μl.

The reaction mixture was then mixed with 20 μl loading buffer containing 1x DNA dye (diluted from blue-orange 6x loading dye; Promega, Madison, WI) and 45% formamide and heated at 95°C for 5 min. The samples were then resolved by sequencing gel electrophoresis (Model S2, Life Technologies, Rockville, MD) at 50°C containing 20% polyacrylamide and 7 M urea. Radioactivity in the incised oligonucleotides was quantified by exposing the gel to x-ray films and measuring the band intensities using an imager (Chemigenius Bioimaging System) with quantification software (Syngene Inc., San Diego, CA).

### 5'- and 3-' tyrosyl DNA phosphodiesterase activity assay in MCF-7 whole cell extract

Two 10 cm plates with 100% confluent MCF-7 cells were harvested and resuspended in 1 ml buffer (40 mM Tris-HCl, pH 7.5, 100 mM NaCl, 0.1% Tween-20, 1 mM DTT, 10% glycerol, and 1 × protease inhibitor [Complete EDTA-free protease inhibitor cocktail tablet, Roche Diagnostics, IN]). They were sonicated (2 × 5 s) on ice at full power using a Braun-Sonic U with a 10 minute gap between the two pulses. Then the whole cell extract was centrifuged for 10 mins in 13000 rpm at 4°C. The supernatant was collected and protein concentration was measured by Biorad protein assay kit (Biorad, CA). We used 1 μg of whole cell extract with 1 nM of 5-sub oligonucleotide in the presence of different concentration of MgCl2 and EDTA for 10 min at 37°C in an assay buffer (50 mM Tris-Cl, pH 7.5, 1 mM DTT, 50 mM KCl and 100 μg/ml BSA) in a total volume of 20 μl.

The substrate and product were analyzed as described in the previous section.

[**Section2**, ID = **Sec12**]

### 5'- and 3-' tyrosyl DNA phosphodiesterase activity assay in deyolked zebrafish embryo extract

Zebrafish were raised, maintained, and crossed as described [28]. All procedures were in accordance with NIH guidelines on the care and use of animals and were approved by the Georgetown University Institutional Animal Care and Use Committee Protocol # 08-019.

Briefly, chorions were removed, in batches of 50 embryos, by limited digestion in 1 mg/ml pronase, followed by three rinses in ice cold Ringer's solution. The embryos were then transferred to cold Ringer's, containing 1 mM EDTA and 0.3 mM phenylmethylsulfonylfluoride (PMSF), and deyolked by titrating through a 200 ul micropipette tip. The deyolked embryos were rinsed in Ringer's solution and stored at -70°C.

Extracts were prepared from wild-type embryos at 24 hours post fertilization (hpf) following the protocol for dechorionating and deyolking embryos [28].

Protein concentration was measured by Biorad protein assay kit (Biorad, CA). We used 2-4 μg of embryo extract with 1 nM of 5-sub oligo in the presence of different concentrations of MgCl_2 _and EDTA for 15 min at 37°C in an assay buffer (50 mM Tris-Cl, pH 7.5, 1 mM DTT, 50 mM KCl,, 15 nM nonspecific single-stranded DNA and 100 μg/ml BSA) in a total volume of 20 μl.

The substrate and product were analyzed as described in the previous section.

## Abbreviations

DSB repair: Double strand Break repair; AP: Apurinic/apyrimidinic; MPG: N-Methylpurine DNA glycosylase; TDP1: 3'- Tyrosyl-DNA phosphodiesterase 1; TDP2: 5'- Tyrosyl-DNA phosphodiesterase 1; hTDP2: human 5'- Tyrosyl-DNA phosphodiesterase 1; hNTH1: Human endonucleases III; APE1: Apurinic/apyrimidinic endonuclease; TDG: Thymine DNA glycosylase; TopI: Topoisomerase I; TopII: Topoisomerase.II.

## Competing interests

The authors declare that they have no competing interests.

## Authors' contributions

SA performed the majority of the experimental work. SKK helped in all the experiments. TMK helped with cell extract preparation. SB and PVM purified the protein. EG assisted in deyolked zebrafish embryo extract experiment. SKK, TMK, SB, PVM, JW, SB, TS and AU assisted in manuscript preparation with critical reading and scientific input. SA supervised, revised, and finalized the project overall, as well as wrote the manuscript. All authors read and approved the final manuscript.
